# Ultrasonographically detected gallbladder polyps: A reason for concern? A seven-year follow-up study

**DOI:** 10.1186/1471-230X-8-41

**Published:** 2008-09-15

**Authors:** Wolfgang Kratzer, Mark M Haenle, Andrea Voegtle, Richard A Mason, Atilla S Akinli, Klaus Hirschbuehl, Andreas Schuler, Volker Kaechele, the Roemerstein study group

**Affiliations:** 1 Zentrum für Innere Medizin, Klinik für Innere Medizin I, Universitätsklinikum Ulm, Robert-Koch-Str. 8, 89081 Ulm, Germany; 2 University Hospitals of Cleveland Case Medical Center, Case Western Reserve, University School of Medicine 11100 Euclid Avenue, Cleveland, Ohio 44106, USA; 3 Zentralklinikum Augsburg, Abteilung Innere Medizin I, Stenglinstr. 2, 86156, Augsburg, Germany; 4 Helfensteinklinik Geislingen, Medizinische Klinik, Eybstr. 16, 73312 Geislingen, Germany

## Abstract

**Background:**

The management of coincidental detected gallbladder polyps (GP) is still nebulous. There are few published data regarding their long-term growth. Objective of the present study was to investigate the prevalence and growth of gallbladder polyps in a survey of unselected subjects from the general population of a complete rural community.

**Methods:**

A total of 2,415 subjects (1,261 women; 1,154 men) underwent ultrasound examination of the gallbladder, in November 1996 as part of a prospective study. Subjects in whom GP were detected at the initial survey underwent follow-up ultrasound examinations after 30 and 84 months.

**Results:**

At the initial survey gallbladder polyps were detected in 34 subjects (1.4%; females: 1.1%, range 14 to 74 years; males: 1.7%, range 19 to 63 years). Median diameter was 5 ± 2.1 mm (range 2 to10 mm) at the initial survey, 5 mm ± 2.8 mm (range 2 to 12 mm) at 30 months and 4 ± 2.3 mm (range 2 to 9 mm) at 84 months. At the time of first follow-up no change in diameter was found in 81.0% (n = 17), reduction in diameter in 4.8% (n = 1) and increase in diameter in 14.3% (n = 3). At the time of second follow-up no increase in polyp diameter was found in 76.9% (n = 10) and reduction in diameter in 7.7% (n = 1). No evidence of malignant disease of the gallbladder was found.

**Conclusion:**

Over a period of seven years little change was measured in the diameter of gallbladder polyps. There was no evidence of malignant disease of the gallbladder in any subject.

## Background

The development and refinement of diagnostic imaging modalities such as computed tomography (CT), magnetic resonance imaging (MRI) and ultrasonography (US) and their widespread application have led to an increase in the coincidental diagnosis of gallbladder stones and gallbladder polyps [1,2]. As a result, clinicians are ever more frequently confronted with the question of how to proceed in cases of coincidentally discovered gallbladder polyps. The appropriate management of these entities remains controversial [3-5].

Gallbladder polyps represent a heterogeneous group of changes in the gallbladder wall and include entities such as cholesterol polyps, inflammatory polyps, adenomas, leiomyomas and lipomas [6]. The prevalence of gallbladder polyps is reported in the range of 0.3–9.5%, depending on the population studied and on the study design. Prevalence figures in European studies fall in the range of 1.0–4.8%, which is lower than reported in Southeast Asian populations [7-15]. In surgical and pathological studies, the prevalence of gallbladder polyps ranges from 0.004 to 13.8% [16].

To date, only a few studies have investigated the growth behavior of gallbladder polyps in follow-up [9,13,17-21]. Follow-up studies in cross sectional random samples have not been published. The majority of available data derive from surgical or ambulatory patients, or were conducted as part of preventive health measures (table 1) [13,17,18,21].

**Table 1:** Studies of the natural progression of gallblader polyps during follow up of patients and/or subjects


**Author Year**	**Country**	**Population**	**n**	**Method**	**Follow-up Period**	**Patients undergoing surgery**		**Changes in polyp diameter during the observation period**			

						N		Reduction	Disappearance	Increase	Unchanged

Eelkema 1962	USA	Patients	113	Cholecystography	15 years						
Moriguchi 1996	Japan	Outpatients	109	Ultrasound	5 years	4	No carcinoma	1.9%	1.9%	11.7%	84.5%
Shinkai 1988	Japan	Patients	60	Ultrasound	Average 22 months	9	No carcinoma	No statistically significant change	No statistically significant change	No statistically significant change	No statistically significant change
Heyder 1990	Germany	Patients: abdominal screening in a surgical population	92	Ultrasound	Average 9 months	2	No carcinoma	No data	13%	6,5%	No data
Collett 1998	New Zealand	Diabetics and healthy controls	564	Ultrasound	2 years – 30 patients 5 years – 22 patients	0	No surgery	No data	No data	No data	No data
Sugiyama 2000	Japan	Surgical patients	125	Ultrasound/Endo-ultrasound	Average 2.6 years	3	No carcinoma	4%	1.6%	7.2%	87.2%
Csendes 2001	Chile	Surgical patients with dyspeptic symptoms or routine examination	98	Ultrasound	Average 5.9 years Range 24–144 months	14	No carcinoma	2%^1^/^2 ^7%	18%^1^/^2^10%	5% ^3^	53%^1^/^2 ^72%

^1 ^after 8 years; ^2 ^after 12 years; ^3 ^after 4 years

Objective of the present study, conducted as part of a complete prospective sonographic survey of a rural population [22,23], was to determine the prevalence of gallbladder polyps and their growth behavior in long-term follow-up.

## Methods

In November and December 1996 we conducted a prospective epidemiological study on the prevalence of alveolar echinococcosis in Römerstein, a rural community in southwestern Germany. As part of this study, the population was also examined for gallbladder polyps [22,23]. Subjects were informed of the additional examination of the gallbladder at the time of the examination itself in order to minimize bias related to a potentially higher response rate on the part of inhabitants with upper abdominal complaints or disorders of the gallbladder. The present study was conducted in accordance with the principles of the Helsinki Declaration and was approved by the ethics commission of Ulm University.

### Ultrasound examinations

All inhabitants aged six years and older were asked to present for examination after a four-hour fasting period. All study participants underwent ultrasound examination of the gallbladder at which gallbladder size in three axes, the gallbladder wall and gallbladder lumen were assessed. In cases of inconclusive findings regarding differentiation between gallbladder stones and gallbladder polyps, patients were examined in standing position.

The initial ultrasound examinations in 1996 were carried out by 8 assistants of the University Hospital of Ulm in 4 cubicles, where examinations were performed simultaneously. All personnel had been trained by the same experienced ultrasound examiner before the study, and this examiner was present in the examination room to provide a second opinion in cases in which the primary examiner could not give a definite diagnosis. The ultrasound examinations in 1999 and 2003 were all performed by the same experienced examiner.

The diagnosis of gallbladder polyps was made on the basis of the following criteria: hyperechoic structures without acoustic shadow that projected from the gallbladder wall into the gallbladder lumen and were either pedunculate or broad based; unequivocal visualization in two planes (longitudinal and in cross-section); no change in position of the wall change secondary to change in subjects' position; unremarkable gallbladder wall; unequivocal differentiation between a gallbladder septum and a gallbladder polyp. The diagnosis of "gallbladder polyp" was made only in cases fulfilling all the above criteria.

Ultrasound examinations were performed using three different types of ultrasound scanners (two units of the type ATL 800 and one ATL 9 HDI, manufactured by ATL Ultrasound Medical Systems, Bothell, WA, USA, each with either a 3.5–5 MHz or 4–7 MHz convex transducer head; and a Siemens Sonoline 400, manufactured by Siemens AG, Erlangen, Germany with a 5 MHz convex transducer head) by trained examiners working under supervision. The number of polyps and the diameter in millimeters of the largest polyp were then documented.

### Follow-up 1999

Subjects diagnosed with gallbladder polyps at the initial survey in 1996 were sent a written invitation in May 1999 for follow-up examination. Follow-up examinations were conducted 30 months after the initial survey from May 25^th ^to June 5^th ^1999. Of 34 subjects diagnosed with gallbladder polyps, 31 (91%) accepted the invitation. Two subjects did not respond to the written invitation, while the third refused the follow-up examination for personal reasons.

The ultrasound examinations were conducted using a Philips ATL HDI 5000 scanner with a 2–5 MHz convex transducer head. An increase or decrease in the diameter of the polyp was defined as a size change of greater than 2 mm.

### Follow-up 2003

The second follow-up examination took place in October and November 2003 at 84 months after the initial survey. With the help of the Civil Registry Office of Römerstein, all 31 subjects participating in the first follow-up examination in 1999 were located. It was discovered that four individuals had moved, while one participant had died. The remaining 26 participants were sent a written invitation and were also contacted by telephone. Subjects were given the choice of being examined either at the University Hospital of Ulm or in Römerstein (school center). In order to maximize the response rate, subjects were also offered the option of being examined at home using a portable ultrasound scanner. Three subjects came to Ulm, while 14 were examined in Römerstein (school center) and five were examined in their homes. Examinations in Ulm and Römerstein were again conducted using a Philips ATL HDI 5000 scanner with a 2–5 MHz and 4–7 MHz convex transducer heads. Subjects examined in their homes were scanned using a portable SONOACE My Sono 201 scanner with a 2–5 MHz convex transducer head.

Three of the 26 subjects had undergone cholecystectomy in the intervening period. One further subject refused to participate in the follow-up examination for personal reasons. Thus, of the remaining 26 subjects, 22 (64.7%, n = 34; 14 males, eight females) participated in the second follow-up examination. Nine subjects could not be examined at follow-up: four had moved, one had died and three had undergone cholecystectomy.

### Statistics

Because of the small number of subjects, data were analyzed descriptively.

## Results

Of the total 3,841 registered inhabitants of the community of Römerstein six years of age or older, 66.6% (n = 2,560) participated in the initial survey in 1996. Excluded from the study were 145 initial respondents (5.7%), of whom 82 (3.2%) had undergone prior cholecystectomy. Sixty-two subjects (2.4%) were excluded because of inability to adequately visualize the gallbladder, while one subject (0.04%) was found to have both a gallbladder stone and a gallbladder polyp. At the initial examinations in 1996, gallbladder findings suggestive of malignancy were not identified in any subject.

### Prevalence

**Figure 1:**
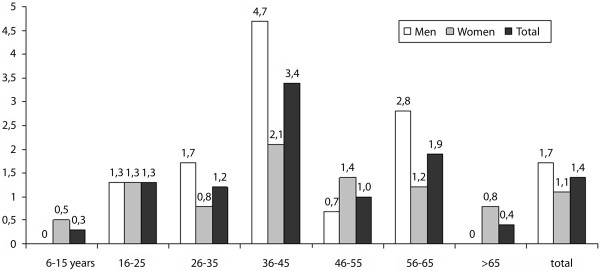
Gallbladder polyps: prevalence in percent (%) in relation to age and sex at the initial survey in 1996.

The study population consisted of n = 2,415 subjects (1,261 women, median age 41.5 years, range 14–74 years; 1,154 men, median age 39 years, range 19–63 years). Sonographic criteria for gallbladder polyps were documented in 1.4% of subjects (n = 34; 1.1% in females; 1.7% in males). The highest prevalence of gallbladder polyps was observed in the group of subjects 36–45 years of age (females, 2.1%; males, 4.7%; figure 1). In the same study population, the prevalence of gallbladder stones was 7.8% (10.5% in females, 4.9% in males; 23).

### Number and diameter of polyps

Data on polyp size are limited to n = 31 subjects because documentation of the diameter of polyps was not available in three subjects. The diameter of the largest polyp was 5 mm or less in 20 of 31 subjects (64.5%) and between 6 mm and 10 mm in 11 of 31 subjects (35.5%). No polyps were larger than 10 mm. Median polyp diameter was 5 mm ± 2.1 mm (range 2–10 mm) for all subjects; 4 mm ± 2.2 mm (range 2–9 mm) in females; and 5 mm ± 2.1 mm (range 4–10 mm) in males. Of the total 34 subjects with identified polyps, 85.3% had solitary polyps, while 14.7% had multiple polyps (2 polyps in two subjects, 3 polyps in two subjects, more than 5 polyps in one subject). All polyps were pedunculate. Broad-based polyps were not observed in any subject

### Follow-up 1999

**Figure 2:**
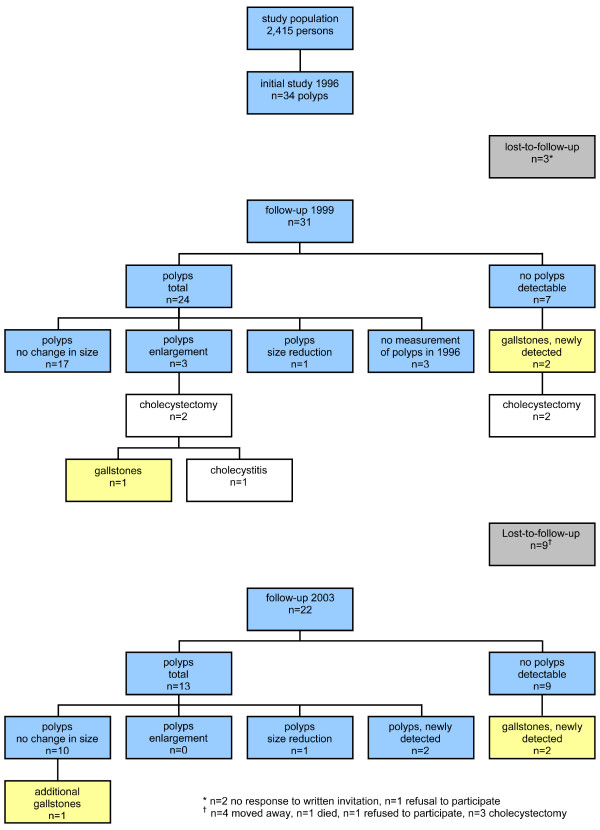
Initial study population in 1996 and results and ultrasound findings in 1999 and 2003.

Of 34 subjects with identified gallbladder polyps at the time of the initial survey, 31 (91.2%; 13 women, 18 men) presented to the first follow-up examination. At the time of the first follow-up examination, polyps were again identified in 24 subjects (77.4%, n = 31). Polyps were no longer visualized in seven subjects. Among these seven subjects, two female subjects, however, had newly diagnosed gallbladder stones (figure 2). Median polyp diameter was 5 mm ± 2.8 (range 2–12 mm). Polyp diameter was less than 10 mm in 91.7% (22/24) of cases and less than 5 mm in 45.8% (11/24) of cases. Two polyps (2/24; 8.3%) were larger than 12 mm. In 81.0% (17/21) of cases, polyp size remained constant, while, in one subject (1/21; 4.8%), polyp diameter decreased and in three subjects (3/21, 14.3%), growth progression was observed (figure 2). In one subject, polyp diameter had doubled from 4 to 8 mm. Unfortunately, this subject left the community shortly after the first follow-up examination, preventing further follow-up. Examination of the two other subjects revealed size increase from 9 to 12 mm. Because of polyp size greater than 10 mm, these patients had been advised to consider cholecystectomy and subsequently did undergo the procedure. Histopathological studies in one subject revealed multiple gallbladder stones in an otherwise unremarkable gallbladder, while, in the second, cholecystitis of moderate severity was diagnosed. These three subjects with increases in polyp diameter did not report upper abdominal complains, nausea or vomiting, or intolerance of fatty foods; two, however, reported infrequent heartburn.

### Follow-up 2003

In the remaining 22 subjects, polyps were no longer visualized in nine persons. In three of these subjects, the first follow-up examination in 1999 had also failed to visualize polyps; follow-up in 2003 showed that no new sonographically visualized polyps had developed in the four-year interval in these subjects. Among the remaining four subjects without polyps, two were found to have developed gallbladder stones in the interval. In one other female subject, the findings of cholecystolithiasis from the first follow-up examination in 1999 was confirmed (figure 2).

Polyp diameter remained constant in 76.9% (10/13) subjects. In one subject (1/13; 7.7%) a polyp decreased in size. Two subjects (2/13; 15.4%), in whom the first follow-up examination in 1999 had failed to visualize the polyps found at the initial survey in 1996, again met sonographic criteria for the diagnosis of gallbladder polyps. A comparison of median polyp size over seven years suggests that their diameter does not significantly change. In all examinations, females had a smaller polyp diameter than did males. Thickening of the gallbladder wall was not observed in any subject. Median diameter of polyps for all subjects was 4 mm ± 2.3 (range 2–9 mm), for women, 4 mm and for males, 5 mm. For the whole population, 53.8% (7/13) of polyps were smaller than 5 mm, while 46.2% (6/13) showed a diameter between 6 and 9 mm.

## Discussion

### Prevalence study

The prevalence of gallbladder polyps in our population sample was 1.4% (males, 1.7%; females 1.1%), which corresponds to prevalence data published by Özmen et al. for Great Britain and by Heyder et al, for Germany [7,9]. Our data were collected as part of a complete survey of a rural population with a response rate of 66.6% [22,23]. To date, the only available data regarding the prevalence of gallbladder polyps in a representative population sample were published by Jørgensen et al., who reported a prevalence of 4.6% for males and 4.3% for females [8]. Prevalence data from larger, non-surgical patient populations derive from preventive medical studies or from selected populations in Japan and Taiwan, with prevalences in the range of 5.3–9.5% [10-14]. All studies show a predominance of males for the development of gallbladder polyps, compared to a female predominance for the development of gallbladder stones [10-14,23,24]. In our study, the peak age for first manifestation of gallbladder polyps lay between 36 and 45 years. In this age group, the respective prevalences for males and females stood at 4.7% and 2.1%, respectively. Segawa and Lin reported the highest prevalence for both sexes in the fourth decade of life [10,15]. Similarly, Okamoto and Jørgensen found the highest prevalence in males in the fourth decade, but in the fifth and sixth decades in females [8,10,11,15].

### Follow-up study

To date, no data on the follow-up of gallbladder polyps derived from studies of representative population samples have been published. Of published studies, the majority used diagnostic ultrasound, while Eelkema et al. analyzed data obtained by cholecystography and Sugiyama et al. examined some of their patients using endosonography. Most subjects were surgical patients, although Collett et al. report on diabetics and healthy controls [9,13,14,17-21]. The average follow-up periods of the available studies range from nine months to 15 years. In most instances, patients were re-examined at intervals of six or 12 months. Similar to the study by Collett et al., we examined our subjects at established follow-up dates of three and seven years [19].

All gallbladder polyps diagnosed at the initial survey were 10 mm or less in diameter. A similar distribution was reported by Moriguchi et al., who reported diameters 5 mm and less in 57%, 6–9 mm in 37% and 10 cm and above in only 6% [13]. Jørgensen et al. and Csendes et al. reported diameters < 5 mm in even higher percentages of subjects, namely 85% and 80%, respectively, and did not, in their respective populations, identify any polyps larger than 10 mm in diameter [8,17]. The median polyp diameter in our initial survey was 5 mm ± 2.1 mm (range 2–10 mm), which was comparable to data reported by Shinkai et al. at 4.8 mm ± 2.9 mm. In the study reported by Collett et al., the average initial diameter was 3.9 mm. Heyder et al., however, reported larger diameters at 6 mm (range 2–15 mm) [9,19,20].

**Figure 3:**
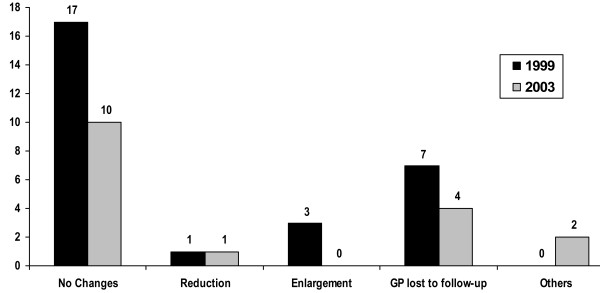
Changes in gallbladder polyps (GP) between 1996 and 1999 and between 1996 and 2003.

With respect to diameter, the majority of polyps showed no change at either the first (1999) or second (2003) follow-up examination. In 1999, polyp diameter remained constant in 81% (17/21) of subjects, with one person (5%) exhibiting reduction in the size of his polyp and three persons (14%) showing size progression (figure 3). A comparison of size progression between 1999 and 2003 shows that 91% (10/11) of polyps remained constant, one became smaller (1/11; 9%) but none became larger. Over the entire 84-month period 62% (8/13) of polyps showed no change in size, 15% (2/13) became smaller and 23% (3/13) became larger. Thus, our results lie between data published by Moriguchi and Sugiyama on the one hand and those of Csendes on the other [13,17,18]. Over an observation period of five years, Moriguchi et al. found an increase in polyp diameter in 11.7% (12/103), while in 84.5% (87/103), polyp size remained constant. Conversely, Csendes et al., who followed subjects for an average 71%, found no change in polyp diameter in 50% of subjects while an increase or decrease in diameter was observed in 25% each (table 1) [13,17].

At the first follow-up examination, 30 months after the initial survey and with a response rate of 91%, polyps were visualized in only 77% (24/31) of subjects; at the second follow-up examination, 84 months after the initial survey and with a response rate of 65%, polyps were identified in 41.9% (13/31) of subjects. The proportion of polyps that were no longer detected at follow-up examinations was lower in reports by other authors [13,17-19].

Of the three subjects in our study who exhibited polyps with size progression, two underwent cholecystectomy. The histopathological findings in one patient revealed multiple gallbladder stones in an otherwise unremarkable gallbladder. In all, 19.4% (n = 6) of all structures initially identified as polyps were identified as gallbladder stones at follow-up. Similar results have been reported from surgical series [6,4,25,26].

A possible reason for the false-positive ultrasound findings might be that polyps may form the site of origin in the development of gallbladder stones [6,27].

The histopathological findings in the second subject who had undergone cholecystectomy due to size progression revealed cholecystitis of moderate severity, which, at the time of the ultrasound examination, may have been polypoid in appearance [27,28]. A further reason for the failure to demonstrate polyps at histopathological examination could be that polyps may be destroyed by the mechanical action of the gallbladder wall.[8] This may also be an explanation for the observation in our study that the prevalence of gallbladder polyps among women and in advanced age is lower, while, at the same time, in this subsample, the prevalence of gallbladder stones is higher to a statistically significant extent. [23] Neither Sugiyama nor Moriguchi nor Csendes reported the development of gallbladder stones at follow-up [17,18,20].

One of the limitations of our study is the small number of only 34 subjects with polyps visualized at ultrasound. One reason may relate to the technically less advanced ultrasound scanners used at the initial survey compared with those used at follow-up. The portable ultrasound scanner used at the second follow-up examination of subjects in their homes may also have limited the strength of the findings and may possible have been the cause of false-negative findings.

## Conclusion

The present study for the first time examined the growth behavior of gallbladder polyps in a representative population sample. In summary, we can conclude that the changes in size in the polyps over an observation period of seven years were slight. Size progression was observed in only three subjects. No evidence of development of malignant disease was observed. Remarkable was the high number of gallbladder stones at follow-up, which initially were diagnosed as gallbladder polyps.

## Competing interests

The authors declare that they have no competing interests.

## Authors' contributions

WK conceived and designed the study, acquired, analysed and interpreted data, and drafted the manuscript. MMH collected, assembled and interpreted the data and drafted the manuscript. AV collected, assembled and interpreted the data and revised the manuscript for important intellectual content. RAM drafted the manuscript. AA analysed and interpreted the data, and revised the manuscript for important intellectual content. KH acquired, analysed and interpreted data, and revised the manuscript for important intellectual content. AS revised the manuscript for important intellectual content. VK helped conceive and design the study, acquired, analysed and interpreted data, and drafted the manuscript. All authors approved the final version of the manuscript. WK is guarantor.

## Pre-publication history

The pre-publication history for this paper can be accessed here:


